# The Relationship Between Illegitimate Tasks and Nurse Work Engagement: The Mediating Role of Workplace Mindfulness

**DOI:** 10.1155/jonm/6928013

**Published:** 2025-09-18

**Authors:** Meiyun Jia, Ke Song, Baojiang Zhang, Xiaona Si, Huiyuan Xue

**Affiliations:** ^1^Nursing Department, People's Hospital of Henan University of Traditional Chinese Medicine, Zhengzhou, Henan, China; ^2^Department of Neurology, People's Hospital of Henan University of Traditional Chinese Medicine, Zhengzhou, Henan, China

**Keywords:** illegitimate tasks, mediating effects, nurses, work engagement, workplace mindfulness

## Abstract

**Aim:** This study aims to explore the relationship between illegitimate tasks and nurses' work engagement, as well as the mediating role of workplace mindfulness in this relationship.

**Design:** This is a quantitative cross-sectional study.

**Methods:** The study was conducted from October to December 2023 across three tertiary hospitals in Central China. Questionnaires on illegitimate tasks, workplace mindfulness, and work engagement were distributed to participating nurses via an online platform, yielding 1458 voluntary responses. SPSS 26.0 was used to analyze the data by *Z*-tests and Spearman rank correlation analysis. The intrinsic mechanism of the effects of workplace mindfulness and illegitimate tasks on nurses' work engagement was examined using AMOS 24.0 statistical software.

**Results:** The total scores of nurses' work engagement, workplace mindfulness, and perceived illegitimate tasks were 34.00 (27.00, 43.00), 75.00 (69.00, 83.00), and 19.00 (14.00, 24.00), respectively. The rank correlation analysis showed that Chinese nurses' work engagement was positively correlated with workplace mindfulness but negatively correlated with illegitimate tasks. And workplace mindfulness was identified as a partial mediator between illegitimate tasks and nurses' work engagement, accounting for 49.1% of the effect.

**Conclusion:** Nurses' work engagement and perceived illegitimate tasks were found to be at moderate levels, while workplace mindfulness received relatively high scores. Additionally, workplace mindfulness acted as a partial mediator between illegitimate tasks and nurses' work engagement.

**Impact:** Nursing managers should actively monitor the work engagement levels of clinical nurses and recognize the negative impact of illegitimate tasks on their engagement. Additionally, fostering mindfulness in daily practices is essential to enhance nurses' work engagement and improve the quality of nursing care.

## 1. Introduction

In the modern healthcare system, nurses serve as essential components of the medical team, playing a crucial role in ensuring patient safety and facilitating recovery [1]. With the emergence of intelligent medical technologies, the scope and responsibilities of nursing practice are continually expanding and deepening, further emphasizing the significance of nurses [2]. However, it is important to note that due to the unique challenges associated with the nursing profession, nurses experience higher turnover rates and mobility [3, 4]. Moreover, staff shortages not only intensify the workload for the remaining nurses but can also lead to a decline in the quality of their work and their ability to maintain patient safety, posing potential risks to patient well-being [5, 6]. Nevertheless, existing research indicates that the effective utilization of nursing staff can provide hospitals with a sustainable competitive advantage, and a critical condition for fully leveraging this advantage is enhancing nurses' work engagement [7]. Therefore, healthcare organizations must not only focus on reducing nurse turnover but also actively promote work engagement among current nursing staff.

Work engagement is a persistent and positive cognitive and emotional state of individuals, and as one of the focal points of positive psychology research, it has increasingly been introduced into the nursing field in recent years [6]. Studies indicate that nurses with high levels of work engagement tend to provide greater attention and patience to patients, which not only enhances patient satisfaction and treatment outcomes [8, 9] but also invigorates their own daily work with energy and enthusiasm. This, in turn, helps to reduce occupational stress [10, 11]. The impact of work engagement on nurses is evident; however, identifying how to enhance this engagement and exploring its antecedents remains an important topic. Scholars have identified several factors influencing nurses' work engagement, including workload, rewards, leadership styles, and mental health [12–14]. Nonetheless, a potential source of stress seems to affect nurses' work engagement—illegitimate tasks [15]. When nurses encounter illegitimate tasks, they may adopt negative coping strategies such as avoidance, resistance, or superficial compliance as a form of self-protection, which can negatively impact their work behavior [16]. Moreover, workplace mindfulness, recognized as a positive psychological resource, has been increasingly applied in nursing environments. Research indicates that mindfulness can effectively buffer the adverse effects experienced by nurses, helping them regulate their emotions and manage stress, thereby preventing occupational burnout and enhancing work efficiency [17]. However, although prior studies have explored the individual effects of illegitimate tasks and mindfulness on nurses' work engagement, there is a scarcity of research examining their combined influence. Moreover, the specific mechanisms underlying the relationships among these three variables remain unclear. Therefore, investigating the intrinsic connections between illegitimate tasks, mindfulness, and work engagement is of considerable importance.

The Job Demands-Resources (JD-R) model is one of the key theoretical frameworks for studying work engagement, proposing that all job characteristics can be classified into two core categories: job demands and job resources [18]. Based on the JD-R model, illegitimate tasks, as a form of job demand, require nurses to expend additional personal resources to complete their work. When nurses face an imbalance characterized by high demands and low resources, their work engagement is likely to decline [16]. Conversely, workplace mindfulness, as a beneficial personal resource, can effectively mitigate the negative effects of illegitimate tasks, thereby enhancing nurses' work engagement [19].

From the perspective of the JD-R theory, the relationships among nurses' work engagement, illegitimate tasks, and workplace mindfulness can be initially elucidated within this theoretical framework. Therefore, the present study is based on the JD-R model and aims to explore the potential structural relationships among nurses' work engagement, illegitimate tasks, and workplace mindfulness and to unravel the “black box” mechanisms linking the three factors, thereby enriching empirical research on the impact of illegitimate tasks on nurses' work engagement. At the same time, it also provides a reference for nursing managers to effectively improve nurses' work engagement and improve the overall quality of nursing.

## 2. Background

### 2.1. Nurse Work Engagement

Work engagement is often described as a psychological state characterized by an individual's heightened enthusiasm, focus, and vitality towards their work [20]. It was initially conceptualized by Kahn [21], who proposed that the relationship between individuals and work is dynamic and dialectical and defined work engagement from physiological, cognitive, and emotional perspectives as “the process by which individuals integrate themselves into their work roles through self-regulation.” With the advancement of positive psychology, scholars have provided more detailed definitions of work engagement. The most widely accepted conceptualization comes from Schaufeli et al. [22], who describe work engagement as “a positive, fulfilling, work-related state of mind.” Schaufeli posits that work engagement encompasses not only energy and focus but also includes the attraction to the work and the individual's enthusiasm and perseverance, further categorizing it into three dimensions: vitality, dedication, and absorption [22]. As a crucial aspect of positive psychology in the workplace, work engagement emphasizes the correlation between individuals and their work, providing a new perspective for individuals to maintain a sustained and stable state in their professional roles. Furthermore, relevant research indicates that when individuals are in a state of positive work engagement, they exhibit heightened emotional and cognitive sensitivity, thereby injecting more vitality into their work and further realizing their self-worth in their roles [23, 24]. With the advancement of globalization and information technology in the 21st century, research on work engagement has deepened and expanded into new domains. Today, work engagement is not only a research object of psychology but has also become an indispensable part in organizational management practice. From performance management to employee training, and then to job design, organizations increasingly recognize that enhancing employees' work engagement can not only improve the well-being of employees but also enhance the innovation and market competitiveness of the organization [20, 23, 25]. As nursing is a critical component of the healthcare system, nurses' work engagement is particularly vital for patient health and safety. However, in recent years, due to the complexity of the healthcare environment, nurses are facing increasing challenges, such as high workloads, emotional stress, and staff shortages, all of which adversely affect nurses' work engagement [26, 27]. Relevant research has shown that nurses with high levels of work engagement are better able to communicate with patients and colleagues, thereby reducing the occurrence of medical errors and further improving the quality of patient care and satisfaction [28]. Additionally, high levels of work engagement can also significantly reduce nurse turnover rates, which is essential for stabilizing the nursing workforce structure [29].

### 2.2. Illegitimate Tasks

In organizations, individuals play specific professional roles, which inherently include expectations for acceptable behavior associated with those roles. However, when a particular task contradicts these expectations, it may constitute an offense to work role [30], referred to as “illegitimate tasks.” In recent years, illegitimate tasks have emerged as a new source of stress in the workplace, attracting increasing attention from scholars. For instance, early research by Semmer et al. [31] found that approximately one-third of tasks faced by employees are considered illegitimate. Subsequent studies, including one by Anskär et al. [32] in 2019, found that over a quarter of healthcare personnel in Sweden believed that a significant number of illegitimate tasks existed in their daily work routines. As research progressed, the connotation of illegitimate tasks has been further refined and expanded. Broadly speaking, illegitimate tasks refer to responsibilities that exceed an employee's specific role and contradict their work expectations [30]. Current academia discourse categorizes illegitimate tasks into two dimensions: unnecessary tasks and unreasonable tasks. Unnecessary tasks are those that could have been avoided or are deemed not worth doing, characterized by three aspects: meaningless tasks, tasks that could have been avoided through reasonable arrangements, and tasks that must be completed to meet the requirements of superiors. In contrast, unreasonable tasks refer to those that exceed an individual's professional role or do not align with their expertise, such as completing tasks that do not match one's professional status or responsibilities, or tasks that place the individual in awkward situations [30]. Research indicates that individuals in organizations tend to prioritize their work roles, and when role expectations are threatened or violated, it can lead to increased work-related stress and negative emotions [33]. Based on the JD-R model, illegitimate tasks, as behaviors that exceed the individual's role expectations, are essentially consuming their time and energy to complete noncore activities, thereby occupying valuable work resources [18]. In a high-demand and low-resource imbalance, such tasks not only hinder an individual's ability to achieve work goals but also exacerbate physical and psychological depletion [34]. In an effort to safeguard their professional roles, employees may opt to reduce their work engagement, consequently prompting counterproductive behaviors such as negativity and disengagement [35]. Furthermore, studies show that nurses, as direct contacts with patients, have work behaviors closely related to patient health [36]. Notably, Florence Nightingale, the founder of modern nursing, emphasized as early as 1860 the importance of creating a fair, respectful, and neutral nursing environment to promote nurses' work engagement, further highlighting the significance of assigning reasonable tasks to nurses [37]. Meanwhile, related research has shown that when clinical nurses are assigned tasks beyond their professional scope, they are more likely to exhibit negative work behaviors, which can adversely affect the quality of patient care [36, 38]. Therefore, based on the statement in Sections 2.1 and 2.2, we propose the following hypothesis:

  Hypothesis 1: Illegitimate tasks negatively impact nurses' work engagement.

### 2.3. Workplace Mindfulness

Workplace mindfulness refers to a way and state in which an individual consciously attends to the present moment with openness, acceptance, and nonjudgmental focus, by remaining aware of the current situation without being distracted by thoughts of the past or future [39]. As an ancient concept originating from Buddhist traditions in ancient India, mindfulness serves as a core component of Buddhist teachings and practices, particularly within Zen and Theravada Buddhism. Its original intent was to eliminate desires such as greed, hatred, and delusion by fostering a clear and focused mind [40]. As stated in the Buddhist classic “The Platform Sutra of the Sixth Patriarch,” “Fundamentally there is no Bodhi-tree, Nor stand of a mirror bright, Since all is void from the beginning, Where can the dust alight?” This also embodies the essential nature of mindfulness practice in Buddhism [41]. Over time, mindfulness has gradually transcended its religious roots, becoming a subject of interest and research in psychology and management. In the 1970s, psychologist Kabat-Zinn [40] introduced mindfulness into the medical field, successfully aiding participants in reducing stress and pain, thereby promoting deeper research into mindfulness. At the same time, in today's complex and rapidly changing work environment, individuals increasingly require effective strategies to enhance productivity and maintain their well-being. As a positive intervention, mindfulness offers organizations new perspectives and methods for fostering employee well-being. Currently, mindfulness-based stress reduction training has been gradually adopted by major corporations, and the application of mindfulness is further expanding [42]. Compared to other professions, clinical nurses confront a variety of patient conditions and complex doctor–patient relationships, along with excessive workloads, resulting in heightened levels of psychological stress and occupational burnout. Therefore, there is a pressing need to enhance the focus on nurse's mental and physical health, and mindfulness may serve as an effective approach. According to the JD-R model, mindfulness, as a positive trait and state, can assist clinical nurses in managing their attention and thoughts. By reducing distractions from work-related stressors, mindfulness provides valuable resource protection, thus preventing further depletion of work resources [19, 43]. Furthermore, positive states and emotions have adaptive evolutionary value, and workplace mindfulness can aid clinical nurses in accepting their current circumstances and responding rationally to stress and negative emotions, ultimately enhancing their work engagement [17]. Based on this rationale, we propose the following hypothesis:

  Hypothesis 2: Workplace mindfulness positively influences nurses' work engagement.

### 2.4. Illegitimate Tasks, Workplace Mindfulness, and Nurse Work Engagement

Based on the aforementioned arguments, it is evident that nurses often harbor implicit expectations of task appropriateness, and illegitimate tasks clearly exceed their core job responsibilities or fail to meet professional standards. According to the JD-R model, illegitimate tasks can be viewed as a type of work demand that often occupies nurses' core time or resources. Furthermore, such tasks may convey messages of disrespect and lack of appreciation, leading nurses to experience self-doubt [44]. In such circumstances, nurses are more likely to exhibit resistance and negative behaviors. Prolonged job demands can deplete individual energy, resulting in burnout and adverse health effects, which in turn directly impact nurses' levels of work engagement. The field of positive psychology suggests that when individuals are in a positive state, they tend to focus more attentively on their surroundings [23]. However, workplace mindfulness, regarded as a positive personal resource, may serve as a buffering mechanism that helps individuals manage stress and maintain work engagement. Research indicates that higher levels of workplace mindfulness enable nurses to regulate their responses effectively by distancing themselves from negative events, thoughts, and experiences, thereby reducing rumination in the face of illegitimate tasks and preserving more energy for work [43]. It is important to note that although workplace mindfulness can mitigate the adverse effects associated with illegitimate tasks, it does not entirely prevent the depletion of personal resources, which may ultimately lead to a resource loss spiral. Based on the above arguments, nurses' workplace mindfulness can protect against resource loss to some extent and potentially act as a bridging factor between illegitimate tasks and work engagement. Therefore, we propose the following hypotheses:

  Hypothesis 3: Illegitimate tasks have a negative impact on nurses' workplace mindfulness.
  Hypothesis 4: Workplace mindfulness mediates the relationship between nurses' illegitimate tasks and their work engagement.

## 3. The Study

### 3.1. Aims

This study aims to explore the current status of illegitimate tasks, workplace mindfulness, and work engagement among clinical nurses and to validate the mediating role of workplace mindfulness in the relationship between illegitimate tasks and work engagement.

### 3.2. Design

This study employs a cross-sectional design.

### 3.3. Setting/Participants

This study employed a convenience sampling method to select nurses from three tertiary hospitals in Central China as participants. Inclusion criteria were as follows: (1) possession of a valid nurse qualification certificate from the People's Republic of China; (2) informed consent and voluntary participation. Exclusion criteria included (1) nurses who had been absent from their positions for 3 months or more due to illness, maternity leave, or other reasons; (2) part-time or temporary nurses. The questionnaire was conducted through an online platform (https://www.wjx.cn). With the approval and cooperation of the head of the nursing department of the participating hospitals, the researchers sent the survey link to various clinical departments. The questionnaire was administered on a voluntary and anonymous basis, with the study objectives and instructions for completion clearly outlined on the first page of the survey. Additionally, to mitigate data bias, a pilot presurvey was conducted to assess the validity and reliability of the questionnaire prior to the formal investigation. Following the main survey, responses with a completion time of less than 2 minutes, consistent self-ratings across items, or contradictory answers were excluded. Each IP address was restricted to one submission. The data collection for this study took place from October to December 2023 and was conducted in accordance with the STROBE guidelines.

The sample size calculation for this study was based on the LRT formula developed by Jobst et al. [45], expressed as *T* = (*N* − 1) ∗ *F*. With the root mean square error of approximate (RMSEA) set to 0.05, alpha errors 0.05, and desired power 80%, the required sample size was determined to be 450. To address potential issues such as missing or invalid responses, the original sample size was increased by 20%. Consequently, a minimum of 540 participating nurses was required. A total of 1458 questionnaires were collected in this study, of which 1348 were valid, with a validity rate of 92.46%.

### 3.4. Measures

#### 3.4.1. Demographic Data Questionnaire

The baseline data of the participants were designed by the researchers and included 12 items: age (years), gender, education levels, working years (years), technical title, department, positions, departmental atmosphere, number of night shifts (months), marital status, number of children, and family atmosphere.

#### 3.4.2. Utrecht Work Engagement Scale (UWES)

The Work Engagement Scale, developed by Schaufeli et al. [22], is primarily designed to measure the mental state of individuals at work. This scale comprises 9 items divided into 3 dimensions, namely: vigor (e.g., “At work, I feel bursting with energy”), dedication (e.g., “I take pride in what I do”), and absorption (e.g., “I get completely immersed in my work”). Each item is rated on a 7-point Likert scale, ranging from “0” (*never*) to “6” (*always*). Although the Work Engagement Scale has been extensively utilized across diverse cultural settings, the reliability and validity assessments conducted in this study demonstrated Cronbach's α coefficient of 0.918 for the scale and the discriminant validity of 0.890, with Cronbach's α coefficients of the three subdimensions—vigor, dedication, and absorption at 0.855, 0.909, and 0.862, respectively. This questionnaire has been fully validated for cultural adaptation in the Chinese healthcare context [46, 47].

#### 3.4.3. Bern Illegitimate Tasks Scale (BITS)

The BITS was developed by Semmer [30] and colleagues based on the theory of “stress as self-offense.” Using the BITS, researchers and managers can effectively quantify the frequency and extent of illegitimate tasks experienced by employees at work. This scale comprises two dimensions: unnecessary tasks (e.g., “Is it meaningful to do these?”) and unreasonable tasks (e.g., “Does it put you in an awkward position?”), with each dimension containing four items. The items are rated on a 5-point Likert scale, where scores range from 1 (*never*) to 5 (*often*), with higher scores indicating a more frequent occurrence of illegitimate tasks. In this study, Cronbach's α of the scale was 0.867, and its discriminant and convergent validity was also well validated, which in turn indicated strong adaptability in the surveyed population. This questionnaire has also been widely adopted and validated within the organizational culture context in China [34].

#### 3.4.4. Workplace Mindfulness Scale (WMS)

The WMS was developed by Professor Zheng et al. [48] in 2022. It is designed to assess individuals' awareness and attentiveness to their current work environment. The scale comprises 18 items organized into 3 dimensions, namely, awareness (e.g., “I can immediately notice when unexpected situations arise at work”), attention (e.g., “I maintain full concentration when conversing with others at work”), and acceptance (e.g., “I do not evaluate my thoughts as good or bad while working”), with each dimension containing 6 items. Responses are rated using a 1–5 scoring system, where “1” indicates “*never*” and “5” represents “*always*.” Higher scores reflect a greater perception of workplace mindfulness. In this study, the scale demonstrated a Cronbach's alpha coefficient of 0.921, with the coefficients for the individual dimensions ranging from 0.829 to 0.912. Furthermore, this questionnaire has shown good applicability within the Chinese nursing cultural context [49].

#### 3.4.5. Control Variables

Based on the findings from previous research by Alkorashy and Alanazi [7], this study utilized age and work years as control variables. In this study, age was treated as a continuous variable using its original values and work years as a categorical variable coded as 1, 2, 3, and 4 for ≤ 5 years, 6–10 years, 11–15 years, and > 15 years, respectively.

### 3.5. Statistical Analysis

IBM SPSS Statistics 26.0 and AMOS 24.0 software were used for data analysis. Categorical data were statistically described using frequencies and proportions, while non-normal measurement data were expressed as interquartile ranges (IQRs). Differences in work engagement scores among nurses with varying demographic characteristics were compared using the *Z*-test. Additionally, Spearman correlation analysis was employed to explore the relationships between illegitimate tasks, workplace mindfulness, and nurse work engagement. The internal consistency reliability of the scales was assessed using Cronbach's alpha coefficient. Model fit was evaluated through various indices, including the chi-square test (*χ*^2^), degree of freedom (df), the ratio of chi-square to degrees of freedom (*χ*^2^/df), Comparative Fit Index (CFI), Tucker–Lewis Index (TLI), RMSEA, and standardized root mean square residual (SRMR). The Bootstrap method (with 5000 resampling iterations) was used to test the significance of the mediating effects. In this study, statistical tests were two-sided, and differences were considered significant at a *p* value of < 0.05.

### 3.6. Ethics Statement

This study was conducted in accordance with the Declaration of Helsinki and strictly adhered to principles of privacy and confidentiality. All data were utilized solely for scientific research purposes, and approval was obtained from the Ethics Committee of the People's Hospital of Henan University of Traditional Chinese Medicine (Approval Code: 2023011162).

## 4. Results

### 4.1. Common Method Bias Detection

To ensure the validity and accuracy of the survey results, Harman's single-factor test was employed in this study to conduct an unrotated exploratory factor analysis of the data. The first extracted factor accounted for 35.59% of the variance, which is below the critical threshold of 50%. This indicates that no significant common method bias was detected in the data [50].

### 4.2. Basic Characteristics

Among the 1348 valid participants, 95.70% were female, with the majority being clinical nurses aged 40 and below (89.98%), and 75% of the nurses were married. In the univariate analysis of nurses' work engagement scores, it can be found that there were statistically significant differences in the demographic factors, including nurses' age, gender, work years, department, and positions. Additionally, it was found that nurses with greater years of experience tended to have higher work engagement scores (Table 1).

### 4.3. Scores of Nurse Work Engagement, Workplace Mindfulness, and Illegitimate Tasks

The results of this study indicated that the median total scores for work engagement, workplace mindfulness, and illegitimate tasks among nurses were 34, 75, and 19, respectively, which were mostly at moderate to high levels. Detailed scores for each dimension of the questionnaires are presented in Table 2.

### 4.4. Correlation Among Nurse Work Engagement, Workplace Mindfulness, and Illegitimate Tasks

The results of the Spearman correlation analysis indicate a positive correlation between workplace mindfulness and nurse work engagement. Conversely, a negative relationship was observed between illegitimate tasks and both work engagement and workplace mindfulness (Table 3).

### 4.5. The Mediating Effect of Workplace Mindfulness Between Illegitimate Tasks and Work Engagement Among Clinical Nurses

**Figure 1:**
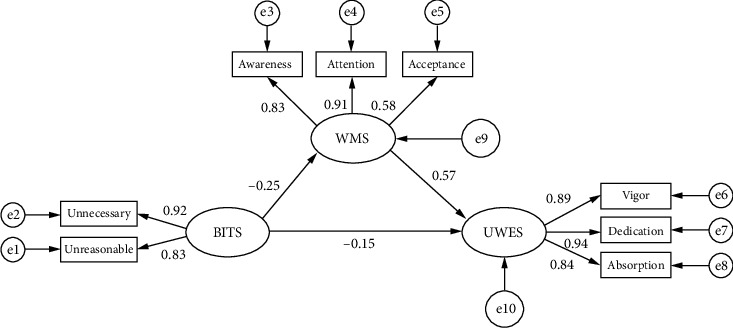
A model of the mediating effect of workplace mindfulness between illegitimate tasks and nurse work engagement.

Based on the aforementioned correlation analysis results and theoretical review, this study constructed a structural model with illegitimate tasks as the independent variable, workplace mindfulness as the mediating variable, and nurse work engagement as the dependent variable, and assessed its fit degree. The fit indices of the model were as follows: CMIN = 185.405, DF = 29, CMIN/DF = 6.393, Goodness-of-Fit Index (GFI) = 0.973, Adjusted Goodness-of-Fit Index (AGFI) = 0.949, CFI = 0.982, TLI = 0.972, Normed Fit Index (NFI) = 0.978, RMSEA = 0.063, and SRMR = 0.0493. The model path analysis, along with convergent and discriminant validity, is detailed in Tables 4 and 5. It is noteworthy that the CMIN/DF value was relatively high, which may be attributed to the large sample size. Additionally, the Bollen–Stine bootstrap correction test yielded a *p* value of < 0.05, further supporting this observation [51]. As illustrated in Figure 1 and Table 6, the mediating effect of workplace mindfulness was found to be −0.148, accounting for 49.1% of the total effect value of −0.301. In summary, workplace mindfulness plays a partial mediating role between illegitimate tasks and work engagement among nurses in Central China.

## 5. Discussion

### 5.1. The Current Status of Nurses' Work Engagement

Nurse work engagement refers to the positive and energetic mental state exhibited by nurses in their work. Research has indicated that higher levels of work engagement enable nurses to derive meaning from their roles, invigorate their enthusiasm, and maintain their psychological and physiological well-being [5, 29]. Consequently, enhancing nurse work engagement can strengthen a hospital's competitiveness and alleviate the issues associated with nursing shortages. In this study, nurses' work engagement was at moderate level. This finding aligns with the results from Cai et al. [46] regarding nurse work engagement, highlighting that there is a need to pay attention to and improve work engagement among nurses. The underlying reasons may be related to work stress, social status, and inequities in compensation. Compared to developed countries, the social status of clinical nurses in China is relatively low. The public often perceives the nursing profession as involving only simple, repetitive tasks such as medication administration and intravenous therapy. In contrast to the respect accorded to physicians, nurses are frequently viewed as easier targets for patient and family grievances during medical disputes. This discrepancy in perceived identity and status may lead to a decline in nurses' enthusiasm for their work [52]. Moreover, with the ongoing healthcare reforms in China, nurses' salaries have significantly decreased compared to the past. Given that nursing is a high-pressure profession, if nurses feel they cannot demonstrate their intrinsic value at work or lack external incentives such as fair compensation, they may experience burnout, leading to reduced work engagement as a means of conserving their internal resources [12]. In this study, the lowest scores on the absorption dimension of work engagement further indicate that nurses are not able to actively engage in their work due to their lower perceived social status and high work stress. Therefore, it remains important to actively focus on and enhance the level of nurses' work engagement.

In this study, demographic factor analysis revealed statistically significant differences in nurses' work engagement based on age, gender, working years, departmental atmosphere, number of night shifts, family atmosphere, number of children, positions, and department. As illustrated in Table 1, there is a noticeable trend of increasing work engagement among nurses with advancing age and working years. This may be attributed to the accumulation of work experience, which equips nurses with richer professional knowledge and practical skills, thereby enhancing their understanding of their roles and fostering greater confidence in their work [53]. Additionally, compared with junior nurses, senior nurses are typically in a phase of career stability and tend to demonstrate greater emotional and psychological maturity. This maturity allows them to navigate stressful situations with greater ease and confidence. In terms of department management, nurse leaders are typically more inclined to assign responsibilities related to personnel management, training, and mentorship to senior nurses, rather than younger staff [7]. This evolution of responsibilities not only facilitates the professional development of experienced nurses but also encourages them to pay closer attention to their own job performance. Furthermore, the study found that female nurses scored higher in work engagement compared to male nurses, which may be associated with nurses' occupational self-esteem and sense of achievement. Generally, nursing has traditionally been viewed as a female-dominated profession, which may expose male nurses to negative societal perceptions and stereotypes [54]. Despite the increasing demand for male nurses in the healthcare sector, pressures from traditional perceptions and societal expectations, among others, may still be psychologically troubling, leading to feelings of isolation in male nurses' professional roles. Consequently, this may hinder male nurses from fully engaging in their work or discourage them from pursuing long-term careers in nursing [55]. At the same time, relevant studies have indicated that interpersonal relationships at work can significantly influence an individual's job quality and emotional state [56]. In this study, the scores for work engagement among nurses in a harmonious departmental atmosphere were notably higher, which may be attributed to the fact that a positive work environment fosters trust and collaboration among nurses, allowing them to share the burdens of work-related stress effectively. In a harmonious atmosphere, nurses are likely to feel more at ease, reducing the fear of criticism or blame, which enables them to focus better on their tasks. Meanwhile, the data revealed that the more night shifts nurses experience, the lower their levels of engagement and motivation. Understandably, frequent night shifts not only disrupt the biological clock, resulting in a decline in nurses' sleep quality and causing health problems [57], but also lead to a reduction of the time available for social interactions with family and friends, resulting in a sense of loneliness and social isolation. Prolonged physical and mental exhaustion can further diminish nurses' ability to engage fully in their work [58]. In contrast, nurses with more children and a better family atmosphere have higher work engagement scores. This phenomenon can be explained by the concept of crossover or spillover effects, where the emotional support provided by children and a harmonious family environment enhances nurses' resilience in facing workplace stressors, thereby improving their overall job performance [59]. Besides, nurse managers reported higher levels of work engagement compared to their staff nurse counterparts. This is likely because nurse managers bear greater responsibilities in ensuring team performance and patient care outcomes, which can motivate them to focus more intently on their work [60]. Additionally, nurse managers often have more opportunities to participate in management training and professional development, allowing them to acquire new knowledge and skills, thereby enhancing their professional competence and engagement. It is noteworthy that nurses working in Emergency Department, Intensive Care Unit, and operating rooms reported the lowest levels of work engagement. This may be attributed to the high-stress, fast-paced working environment of these departments. Research has shown that nurses in Emergency Department, Intensive Care Unit, and operating rooms experience greater work-related stress compared to those in general wards, often facing more urgent and demanding tasks [61]. The high-intensity nature of their work can lead to physical and emotional exhaustion. Moreover, the uncertainty regarding patient outcomes and higher mortality rates associated with critical care can place significant emotional strain on nurses, contributing to psychological fatigue and reduced engagement. In summary, factors such as age, gender, years of experience, departmental atmosphere, family environment, and the nature of their department can all influence nurses' levels of work engagement. Therefore, nursing managers should prioritize the psychological well-being of younger nurses, actively foster a harmonious work environment, and implement flexible management strategies and scheduling practices to enhance their motivation and engagement levels.

### 5.2. Current Status of Illegitimate Tasks Among Nurses

From Table 2, it can be observed that the total score for illegitimate tasks among nurses is at a moderate level, which is lower than the findings reported by Kilponen et al. [15]. This discrepancy may be attributed to cultural differences and the assessment tools used. Unlike the egalitarian hierarchical relationships typical in Western cultures, which emphasize equal contractual relationships, traditional Chinese culture places greater importance on interpersonal relationships and face-saving, highlighting a collectivist spirit that prioritizes team needs over individual demands [62, 63]. In high power distance organizational contexts in China, individuals may not openly exhibit negative behaviors in response to illegitimate tasks; instead, they often adopt a more passive approach to manage such situations. Consequently, when Chinese clinical nurses encounter illegitimate tasks from superiors, they are more likely to opt for tolerance or compliance rather than direct confrontation. It is important to note that, despite the lower scores for illegitimate tasks among Chinese clinical nurses compared to other countries, the overall levels remain relatively high. This may be due to the fact that although Chinese clinical nurses are influenced by traditional values that encourage high tolerance levels, they are more willing to accept jobs that align with their roles as societal development progresses and individual awareness increases. Moreover, in the Chinese organizational culture, which emphasizes leadership authority, there exists an asymmetrical power dependency between nurses and their leaders. Leaders often possess absolute power over nurses, resulting in a strong imposition of authority and control that demands unconditional compliance of nurses [64]. Some unreasonable work arrangements may exacerbate emotional and behavioral resistance among nurses, resulting in a dual conflict that affects their overall job satisfaction and engagement [65]. Moreover, within the Chinese healthcare environment, the nursing department plays a crucial role in managing the nursing workforce across hospitals. However, this authority can also have negative repercussions for nurses. The nursing department's significant power can lead to the imposition of tasks on clinical departments without adequate investigation or regard for the clinical realities, ultimately contributing to illegitimate tasks. Frontline clinical nurses, faced with resource shortages and the mandated tasks from the nursing department, may experience resource depletion and develop a sense of resistance. Thus, hospitals should pay attention to the training of nursing managers and establish clear responsibility frameworks. Nursing managers should consider aligning roles and responsibilities when assigning tasks to clinical nurses and actively monitor their psychological well-being while providing appropriate incentives and support to alleviate nurses' dissatisfaction in their work environment.

### 5.3. The Current Status of Nurses Workplace Mindfulness

Interestingly, this study found that clinical nurses exhibited a moderately high level of mindfulness overall. This phenomenon may be linked to the unique characteristics of the nursing profession. Nursing is inherently filled with uncertainty, requiring nurses to frequently navigate complex and urgent medical situations. As a result, they must possess strong focus and emotional regulation skills, which are essential for effectively managing stress and anxiety and improving performance in high-pressure environments—which aligns closely with the tenets of mindfulness [49]. Additionally, clinical nurses often engage in communication and interaction with patients and their families, necessitating stable emotional and cognitive states to better perceive patients' needs and feelings. At the same time, the relatively high levels of mindfulness observed among nurses in this study may also be related to the current economic and employment situation in China. Due to the impact of the COVID-19 pandemic, all works of life in China have been significantly impacted. Although China's economy has been in a continuous upward trend post-pandemic, it may still fall short of previous levels, leading to decreased incomes and rising unemployment rates. In contrast to other industries, although nurses may not enjoy a highly supportive work environment, their employment remains relatively stable [3]. This stability facilitates a psychological state of “living in the moment,” contributing to their overall mindfulness. It is important to note, however, that as this study relies on self-reported data, there may still be instances of social desirability bias, despite the exclusion of invalid responses. Therefore, it is recommended that nursing managers actively monitor the psychological well-being of clinical nurses and provide targeted mindfulness training to support their mental health.

### 5.4. The Mediating Role of Workplace Mindfulness in the Relationship Between Illegitimate Tasks and Work Engagement Among Nurses

The results of this study indicate that illegitimate tasks are negatively correlated with nurses' workplace mindfulness (*r* = −0.237, *p* < 0.01) and work engagement (*r* = −0.277, *p* < 0.01), while workplace mindfulness has a positive facilitating effect on clinical nurses' work engagement (*r* = 0.557, *p* < 0.01). Notably, the introduction of workplace mindfulness significantly reduces the direct negative impact of illegitimate tasks on nurses' work engagement. It is evident that workplace mindfulness serves as a partial mediator in this relationship, with a mediation effect size of −0.148, accounting for 49.1% of the total effect. This indicates that while illegitimate tasks can directly affect nurses' work engagement, they can also mitigate the detrimental effects on engagement through the mediating variable of mindfulness. An analysis of these findings reveals that illegitimate tasks, as a source of negative stress, clearly contradict clinical nurses' work responsibilities and internal expectations [44]. Moreover, illegitimate tasks often require nurses to expend additional time or effort to address them. This high-demand and low-resource state not only depletes their work resources but also exacerbates negative emotional experiences, thereby leading to diminished work motivation. However, research has shown that workplace mindfulness, as a personal resource, can effectively mitigate the adverse effects associated with illegitimate tasks [19]. In practical work, mindfulness can assist clinical nurses in better perceiving their emotional fluctuations and enhancing their self-awareness. When faced with unreasonable tasks, nurses can examine their reactions and decisions through mindfulness, so as to better control their emotions and respond appropriately, which reduces the negative impact of illegitimate tasks on their emotional and behavioral states [17]. Additionally, due to the inherent qualities of workplace mindfulness, it enables nurses to let go of concerns about the past and the future, allowing them to focus their attention on the tasks at hand, ultimately improving their work efficiency and quality. Therefore, workplace mindfulness not only positively predicts nurses' work engagement but also plays a “bridging” role between illegitimate tasks and nurses' work engagement, partially offsetting the negative impact of illegitimate tasks on engagement. Notably, compared to the findings reported by Karahan Kaplan et al. [66], nurses in the present study exhibited a stronger mediating effect. This difference may be attributed to the pronounced collectivist culture and unique sources of occupational stress prevalent in Chinese hospitals. In the Chinese context, nurses may be more sensitive to the negative impacts of illegitimate tasks; however, within a cultural environment that emphasizes respect for authority and obedience, they are less likely to overtly express psychological distress despite experiencing significant internal discomfort, thereby exacerbating their internal stress. Workplace mindfulness, in this regard, appears to buffer these adverse effects and enhances nurses' capacity to effectively manage occupational stress. Therefore, to improve nurses' workplace mindfulness, hospitals should consider culturally tailored training and support programs that address local cultural characteristics, thereby more effectively equipping nurses to cope with the challenges encountered in their work environment.

### 5.5. Implications for Managers

Based on the above discussion, it is recommended that nursing managers fully recognize the negative impact of illegitimate tasks on nurses and actively optimize the work environment and professional support systems. Careful scheduling of nurses' tasks and shifts should be implemented, emphasizing the person-job fit principle during task allocation to ensure an appropriate match between nurses' capabilities and work demands. Hospital leadership should also strengthen standardized operating procedures and supervision mechanisms. By refining and clearly defining nursing operation standards and protocols, nurses can have well-established guidelines to follow when performing their duties. At the same time, effective communication between nurses and management should be encouraged to foster an open and inclusive work atmosphere, enabling nurses to proactively report difficulties and potential risks encountered in their daily work. Furthermore, given the partial mediating role of workplace mindfulness between illegitimate tasks and nurses' work engagement, it is recommended that hospitals actively promote mindfulness-related training and intervention programs. By incorporating courses such as mindfulness meditation, emotion regulation, and stress management, nurses can enhance their self-awareness and emotional regulation abilities. At the same time, to adapt to the fragmented time available to nurses under a shift-based system, nursing managers can adopt the “microtraining” positive thinking practice of 5–10 min, or incorporate mindfulness practices into nursing continuing education credits or professional development programs, and dynamically optimize the training content through regular evaluation and feedback, so as to gradually integrate mindfulness into their work environment, thus achieving the purpose of enhancing their work engagement and overall well-being.

### 5.6. Limitations

This study has several limitations. Firstly, as with most cross-sectional studies, the findings represent only the situation at a certain time point and may not reveal the dynamic relationships over time among illegitimate tasks, workplace mindfulness, and work engagement. Secondly, the data were collected through self-reports from nurses, and even though more authoritative measurement tools were employed and invalid questionnaires were excluded, there may still be issues related to response bias or social desirability. Thirdly, the sample was primarily drawn from several hospitals in China. Although the three participating hospitals represent typical tertiary medical institutions in Central China, disparities in the allocation of medical resources across different regions may lead to variations in task distribution patterns. Moreover, the external validity of the results may be limited due to the cultural environments, management styles, and human resource policies in different countries and hospitals. Lastly, although this study is grounded in the JD-R model and explores nurses' insights into work engagement when faced with illegitimate tasks, it primarily focuses on the individual level and does not delve into other potential moderating or mediating factors that may influence nurses' work engagement and illegitimate tasks, such as leadership styles, job autonomy, and organizational support. Therefore, future research is recommended to adopt multicenter or longitudinal time-series designs to conduct comparative studies across different cultures and regions to explore how varying cultural contexts, hospital levels, and organizational environments influence nurses' work behaviors and psychological responses when addressing illegitimate tasks. Additionally, a mixed-methods design that integrates both quantitative and qualitative data could be employed to explore how factors such as organizational support, leadership style, or personal autonomy influence nurses' work engagement. By constructing a more comprehensive research framework, we can enable a deeper understanding of the multidimensional factors and variability affecting nurses' work engagement, thereby providing a more thorough theoretical explanation of the intrinsic mechanisms among illegitimate tasks, workplace mindfulness, and work engagement.

## 6. Conclusion

This study explored the relationship between work engagement and illegitimate tasks among nurses in Central China and analyzed the mediating role of workplace mindfulness in this relationship. Through an empirical survey of the nursing population, we found that nurses demonstrated a moderate level of work engagement, while illegitimate tasks negatively impacted their engagement. Additionally, workplace mindfulness played a significant mediating role in this relationship, confirming hypotheses H1 to H4. Theoretically, this study enriches the research on nurses' work engagement by grounding our analysis in the JD-R model and examining the interactions among illegitimate tasks, workplace mindfulness, and work engagement. Furthermore, by introducing a mediation model, we elucidated how nurses adjust their psychological states through mindfulness in the work environment, thereby influencing their level of engagement. This not only contributes to the theoretical understanding of nursing work behavior but also sheds light on the “black box” of the relationship between illegitimate tasks and nurse work engagement. In practical terms, this finding can assist nursing managers in recognizing the negative impact of illegitimate tasks on nurses' work engagement. The introduction of workplace mindfulness also offers nursing managers a novel methodological approach to enhance nurses' work engagement. Therefore, based on the research findings, it is recommended that nursing managers prioritize the principle of person-job fit during task allocation to identify and reduce illegitimate tasks at the source, optimize workflows, and appropriately allocate resources, so as to effectively enhance nurses' work efficiency and satisfaction. Additionally, nursing managers should closely monitor nurses' psychological well-being and cultivate mindfulness skills to help them better manage workplace challenges and stress, ultimately improving their emotional states and work performance.

## Figures and Tables

**Table 1:** Work engagement scores of nurses with different characteristics (*n* = 1348).

Variables	*N* (%)	Work engagement	*Z*	*p*
Age (years)			30.052	0.001
≤ 25	111 (8.23)	33.00 (27.00, 42.00)		
26–30	325 (24.11)	32.00 (27.00, 41.00)		
31–35	442 (32.79)	34.00 (27.00, 43.00)		
36–40	335 (24.85)	35.00 (28.00, 43.00)		
> 40	135 (10.02)	38.00 (32.00, 49.00)		
Gender			−1.990	0.047
Male	58 (4.30)	31.00 (27.00, 37.00)		
Female	1290 (95.70)	34.00 (28.00, 43.00)		
Educational levels			2.153	0.341
Junior college	66 (4.89)	33.00 (27.00, 46.00)		
Undergraduate	1271 (94.29)	34.00 (27.00, 43.00)		
Master degree or above	11 (0.82)	29.00 (25.00, 36.00)		
Working years (years)			14.815	0.002
≤ 5	332 (24.63)	33.00 (27.00, 40.75)		
6–10	289 (21.44)	34.00 (27.00, 43.00)		
11–15	453 (33.61)	35.00 (28.00, 43.00)		
> 15	274 (20.33)	35.50 (29.00, 46.00)		
Technical title			4.888	0.087
Primary title	664 (49.26)	34.00 (27.00, 43.00)		
Intermediate title	645 (47.85)	35.00 (28.00, 43.00)		
Senior title	39 (2.89)	35.00 (32.00, 42.00)		
Department			46.582	0.001
Internal Medicine	301 (22.33)	34.00 (28.00, 40.50)		
Surgical	233 (17.28)	34.00 (27.00, 43.00)		
Obstetrics and Gynecology	81 (6.01)	41.00 (34.00, 49.50)		
Pediatrics	99 (7.34)	34.00 (27.00, 42.00)		
Outpatient and other	292 (21.66)	36.00 (29.00, 45.75)		
Emergency, Intensive Care Unit, and operating room	342 (25.37)	32.00 (26.00, 39.25)		
Positions			−3.857	0.001
Head nurse	83 (6.16)	39.00 (33.00, 46.00)		
Nurse	1265 (93.84)	34.00 (27.00, 42.50)		
Department atmosphere			−6.578	0.001
Harmony	1238 (91.84)	35.00 (28.00, 44.00)		
Generally	110 (8.16)	28.00 (24.00, 34.00)		
≥ 2	456 (33.83)	36.00 (29.00, 44.00)		
Number of night shifts (months)			24.642	0.001
0	547 (40.58)	35.00 (28.00, 44.00)		
1–5	312 (23.15)	34.00 (28.00, 44.00)		
6–10	464 (34.42)	33.00 (27.00, 41.00)		
> 10	25 (1.85)	30.00 (21.00, 32.50)		
Marital status			5.521	0.063
Married	1011 (75.00)	35.00 (28.00, 43.00)		
Single	324 (24.04)	33.00 (27.00, 41.00)		
Widowed or separated	13 (0.96)	38.00 (30.50, 50.00)		
Number of children			12.656	0.002
0	443 (32.86)	33.00 (27.00, 41.00)		
1	449 (33.31)	34.00 (27.00, 42.50)		
≥ 2	456 (33.83)	36.00 (29.00, 44.00)		
Family atmosphere			−5.631	0.001
Harmony	1266 (93.92)	35.00 (28.00, 43.00)		
Generally	82 (6.08)	28.00 (23.00, 34.00)		

**Table 2:** Scores of 1348 nurses on illegitimate tasks, workplace mindfulness, and work engagement [*M* (*P*_25_, *P*_75_)].

Items	*M*	*P*_25_	*P*_75_	Minimum	Maximum
Total illegitimate tasks score	19.00	14.00	24.00	8.00	40.00
Unnecessary tasks	10.00	8.00	13.00	4.00	20.00
Unreasonable tasks	8.00	6.00	12.00	4.00	20.00
Total Workplace Mindfulness score	75.00	69.00	83.00	32.00	90.00
Awareness	26.50	24.00	30.00	12.00	30.00
Attention	27.00	24.00	30.00	7.00	30.00
Acceptance	22.00	20.00	25.00	9.00	30.00
Total work engagement score	34.00	27.00	43.00	8.00	54.00
Vigor	12.00	9.00	15.00	3.00	18.00
Dedication	12.00	9.00	15.00	2.00	18.00
Absorption	10.00	8.00	13.00	1.00	18.00

**Table 3:** Correlation analysis of nurses' work engagement with illegitimate tasks and workplace mindfulness (*r*, *n* = 1348).

Items	1	2	3	4	5	6	7	8	9	10	11
1. Unnecessary tasks	1.000										
2. Unreasonable tasks	0.760^∗∗^	1.000									
3. BITS total score	0.945^∗∗^	0.925^∗∗^	1.000								
4. Awareness	−0.191^∗∗^	−0.228^∗∗^	−0.219^∗∗^	1.000							
5. Attention	−0.239^∗∗^	−0.242^∗∗^	−0.253^∗∗^	0.743^∗∗^	1.000						
6. Acceptance	−0.122^∗∗^	−0.145^∗∗^	−0.139^∗∗^	0.478^∗∗^	0.505^∗∗^	1.000					
7. WMS total score	−0.214^∗∗^	−0.238^∗∗^	−0.237^∗∗^	0.848^∗∗^	0.862^∗∗^	0.807^∗∗^	1.000				
8. Vigor	−0.255^∗∗^	−0.240^∗∗^	−0.265^∗∗^	0.487^∗∗^	0.560^∗∗^	0.421^∗∗^	0.575^∗∗^	1.000			
9. Dedication	−0.306^∗∗^	−0.282^∗∗^	−0.312^∗∗^	0.448^∗∗^	0.534^∗∗^	0.392^∗∗^	0.539^∗∗^	0.832^∗∗^	1.000		
10. Absorption	−0.196^∗∗^	−0.156^∗∗^	−0.188^∗∗^	0.351^∗∗^	0.444^∗∗^	0.326^∗∗^	0.435^∗∗^	0.725^∗∗^	0.779^∗∗^	1.000	
11. UWES total score	−0.275^∗∗^	−0.245^∗∗^	−0.277^∗∗^	0.461^∗∗^	0.553^∗∗^	0.410^∗∗^	0.557^∗∗^	0.917^∗∗^	0.943^∗∗^	0.899^∗∗^	1.000

Abbreviations: BITS, Bern Illegitimate Tasks Scale; UWES, Utrecht Work Engagement Scale; WMS, Workplace Mindfulness Scale.

^∗∗^ *p* < 0.01.

**Table 4:** Model parameters of the structural equation of nurses' work engagement status (*n* = 1348).

Path relationships	Unstd.	S.E.	C.R. (*t*-value)	*p* value	Std.	CR	AVE
Unreasonable tasks ← Illegitimate tasks	1.000				0.832	0.870	0.771
Unnecessary tasks ← Illegitimate tasks	1.208	0.095	12.755	< 0.001	0.922		

Awareness ← Workplace mindfulness	1.000				0.828	0.824	0.617
Attention ← Workplace mindfulness	1.130	0.034	33.382	< 0.001	0.911		
Acceptance ← Workplace mindfulness	0.887	0.041	21.803	< 0.001	0.579		

Vigor ← Work engagement	1.000				0.890	0.919	0.792
Dedication ← Work engagement	1.117	0.022	49.944	< 0.001	0.940		
Absorption ← Work engagement	1.056	0.025	41.802	< 0.001	0.837		

Work engagement ← Workplace mindfulness	0.644	0.033	19.494	< 0.001	0.575		
Workplace mindfulness ← Illegitimate tasks	−0.230	0.028	−8.180	< 0.001	−0.252		
Work engagement ← Illegitimate tasks	−0.153	0.029	−5.300	< 0.001	−0.150		

**Table 5:** Structural model discriminant validity.

Variables	AVE	BITS	WMS	UWES
BITS	0.771	**0.878**	0.270	0.253
WMS	0.617	−0.252	**0.785**	0.643
UWES	0.792	−0.295	0.613	**0.890**

*Note:* The upper triangle presents the HTMT matrix, the lower triangle shows the Fornell–Larcker criterion, and the diagonal elements represent the square roots of the AVE values. The bolded values represent the square root of the AVE values. They are highlighted in bold to distinguish the HTMT matrix displayed in the upper triangle from the Fornell–Larcker criteria shown in the lower triangle.

Abbreviations: BITS, Bern Illegitimate Tasks Scale; UWES, Utrecht Work Engagement Scale; WMS, Workplace Mindfulness Scale.

**Table 6:** Confidence interval of mediation effect of model (5000 bootstrap samples).

Model path	Estimate	95% CI		*p*	Boot S.E.	Effect ratio (%)
		LLCI	ULCI			
BITS ⟶ UWES	−0.153	−0.224	−0.076	< 0.001	0.038	50.9
BITS ⟶ WMS ⟶ UWES	−0.148	−0.188	−0.106	< 0.001	0.021	49.1
Total effect	−0.301	−0.376	−0.223	< 0.001	0.039	100

Abbreviations: BITS, Bern Illegitimate Tasks Scale; UWES, Utrecht Work Engagement Scale; WMS, Workplace Mindfulness Scale.

## Data Availability

The data that support the findings of this study are available from the corresponding author upon reasonable request.
